# The Use of Cannabidiol in Metabolic Syndrome—An Opportunity to Improve the Patient’s Health or Much Ado about Nothing?

**DOI:** 10.3390/jcm12144620

**Published:** 2023-07-11

**Authors:** Michał Wiciński, Anna Fajkiel-Madajczyk, Zuzanna Kurant, Karol Gryczka, Dominik Kurant, Monika Szambelan, Bartosz Malinowski, Michal Falkowski, Jan Zabrzyński, Maciej Słupski

**Affiliations:** 1 Department of Pharmacology and Therapeutics, Faculty of Medicine, Collegium Medicum in Bydgoszcz, Nicolaus Copernicus University, M. Curie Skłodowskiej 9, 85-094 Bydgoszcz, Poland; michal.wicinski@cm.umk.pl (M.W.); zuzannakurant@gmail.com (Z.K.); 98.karol@gmail.com (K.G.); dominokurant1@gmail.com (D.K.); monika.szambelan@cm.umk.pl (M.S.); bartosz.malin@gmail.com (B.M.); 2 Department of Medicinal Chemistry, Faculty of Pharmacy, Collegium Medicum in Bydgoszcz, Nicolaus Copernicus University in Toruń, Dr. A. Jurasza 2, 85-089 Bydgoszcz, Poland; m.falkowski@cm.umk.pl; 3 Department of Orthopedics and Traumatology, Faculty of Medicine, Collegium Medicum in Bydgoszcz, Nicolaus Copernicus University, M. Curie Skłodowskiej 9, 85-094 Bydgoszcz, Poland; jan.zabrzynski@cm.umk.pl; 4 Department of Hepatobiliary and General Surgery, Faculty of Medicine, Collegium Medicum in Bydgoszcz, Nicolaus Copernicus University, M. Curie Skłodowskiej 9, 85-094 Bydgoszcz, Poland; maciej.slupski@cm.umk.pl

**Keywords:** cannabidiol, metabolic syndrome, dyslipidemia, obesity, NAFLD

## Abstract

Cannabis-derived therapies are gaining popularity in the medical world. More and more perfect forms of cannabinoids are sought, which could be used in the treatment of many common diseases, including metabolic syndrome, whose occurrence is also increasing. The purpose of this review was to investigate the usefulness of cannabinoids, mainly cannabidiol (CBD), in individuals with obesity, impaired glucose and lipid metabolism, high blood pressure, and non-alcoholic fatty liver disease (NAFLD). We summarised the most recent research on the broad topic of cannabis-derived influence on metabolic syndrome components. Since there is a lot of work on the effects of Δ9-THC (Δ9-tetrahydrocannabinol) on metabolism and far less on cannabidiol, we felt it needed to be sorted out and summarised in this review. The research results on the use of cannabidiol in obesity are contraindicatory. When it comes to glucose homeostasis, it appears that CBD maintains it, sensitises adipose tissue to insulin, and reduces fasting glucose levels, so it seems to be a potential target in this kind of metabolic disorder, but some research results are inconclusive. CBD shows some promising results in the treatment of various lipid disorders. Some studies have proven its positive effect by decreasing LDL and increasing HDL as well. Despite their probable efficacy, CBD and its derivatives will likely remain an adjunctive treatment rather than a mainstay of therapy. Studies have also shown that CBD in patients with hypertension has positive effects, even though the hypotensive properties of cannabidiol are small. However, CBD can be used to prevent blood pressure surges, stabilise them, and have a protective effect on blood vessels. Results from preclinical studies have shown that the effect of cannabidiol on NAFLD may be potentially beneficial in the treatment of the metabolic syndrome and its components. Nevertheless, there is limited data on CBD and NAFLD in human studies. Because of the numerous confounding factors, the conclusions are unclear, and more research in this field is required.

## 1. Introduction

Given the significance of the metabolic syndrome and the health problems it causes, as well as the rapid growth of the market for cannabis-derived medical treatments, we decided to investigate the influence they had on the different components of the metabolic syndrome.

Recently, issues regarding the use of cannabis or its chemical constituents in medicine have become very common. Presently, 64 countries have provisions or guidelines concerning medical cannabis use or cannabinoid pharmaceutical preparation [1]. Its therapeutic effects depend on the type of cannabinoids used. The major substances isolated from Cannabis sativa are: Δ9-tetrahydrocannabinol (Δ9-THC), Δ9-tetrahydrocannabivarin (Δ9-THCV), cannabinol (CBN), cannabidiol (CBD), cannabidivarin (CBDV), cannabigerol (CBG), and cannabichromene (CBC) [2]. Two major phytocannabinoids that receive the most attention are Δ9-THC and CBD—both are in medical use and, despite their similar chemical structure, differ in the effects they exert. Δ9-THC is known for its psychoactive properties, which CBD does not have [3]. This last one is rapidly absorbed by adipose tissue and other organs and passes through the blood-brain barrier into the central nervous system (CNS). Unfortunately, it has low bioavailability after oral administration (6–19%) [4], and consequently, there is a need to look for new formulations with better absorption. In contrast to Δ9-THC, administration of CBD in a wide range of doses by various routes does not cause serious side effects or toxicity in humans [5], therefore it has the potential to be a safe drug for many disorders. Due to their multidirectional activity, for example, anti-inflammatory, antiviral, or neuroprotective, they have become a subject of interest in many medical specialties [6,7]. There are a lot of studies that describe cannabinoids impact on pain management, Parkinson’s disease, spasticity due to multiple sclerosis or paraplegia, epilepsy, ophthalmological diseases, or different kinds of psychiatric disorders [8,9,10,11,12,13,14,15,16]. In recent years, interest in the potential therapeutic properties of CBD in the treatment of cardiovascular [17], cancer [18], metabolic [19], and neurodegenerative [20] diseases has increased.

The recreational use of marijuana is becoming more popular too, and cannabis remains the most widely used drug in the world. According to the United Nations Office on Drugs and Crime World Drugs Report 2022, cannabinoids daily use increased due to their accessibility and cannabis legalisation in some countries. In 2020, more than 4% of the global population between 15 and 64 had used cannabis in past years, meaning that there were about 209 million cannabis users in the world [1].

In view of the above, it seems interesting how cannabinoids affect human health. More specifically, how their use relates to metabolic syndrome (MetS), whose prevalence in the global population becomes more frequent as well [21]. According to the International Diabetes Federation, as much as one quarter of the world’s population met criteria for metabolic syndrome [22]. The prevalence of it depends on the region—rural or urban area—the composition of the population—ethnicity, sex, age, race—and accepted criteria for the diagnosis of MetS. On average, it ranges from <10 to 80%. The prevalence of this condition is particularly high in all Western societies, and the likely reason for this is the high prevalence of central obesity [23]. Metabolic syndrome is not included in the International Classification of Disease. The diagnosis according to the following organisations: IDF (International Diabetes Federation), NHLBI (National Heart, Lung, and Blood Institute), AHA (American Heart Association), WHF (World Heart Federation), IAS (International Atherosclerosis Society), and IASO (International Association for the Study of Obesity) is made when at least 3 out of 5 criteria are present:

(1) Increased waist circumference (depends on country of origin and ethnic group—in the European Caucasian population, ≥80 cm in women and ≥94 cm in men);
(2) Fasting triglycerides > 1.7 mmol/L (150 mg/dL) or treatment for hypertriglyceridemia;
(3) Fasting HDL-C < 1.0 mmol/L (40 mg/dL) in men and <1.3 mmol/L (50 mg/dL) in women or treatment for this lipid disorder;
(4) Systolic blood pressure ≥ 130 mmHg or diastolic blood pressure ≥ 85 mmHg, or treatment of previously diagnosed hypertension;
(5) Fasting plasma glucose ≥ 5.6 mmol/L (100 mg/dL) or medication for type 2 diabetes.

Moreover, they distinguished additional conditions of the metabolic syndrome as the consequences of obesity, like impaired kidney function, obstructive sleep apnea, heart failure with preserved ejection fraction, fatty liver disease, polycystic ovary syndrome, hyperuricemia, chronic inflammation, sympathetic activation, and tachycardia [24].

In this review, we summarised how the endogenous cannabinoid system and cannabinoids are associated with metabolic syndrome parameters, particularly focusing on cannabidiol and its impact on obesity, glucose metabolism, plasma lipids, blood pressure, and non-alcoholic fatty liver disease.

## 2. The Endocannabinoid System—Biological Effect of Cannabinoids

The cannabinoids interact mainly with the metabotropic CB1 and CB2 receptors—one of the main components of the endocannabinoid system (ECS). CB1R and CB2R are members of the G protein-coupled receptor (GPCR) family and are distributed widely throughout the body. Activation of these receptors inhibits adenylyl cyclase activity, which affects the cytoplasmic concentration of cyclic adenosine monophosphate (cAMP) and the resulting stimulation of multiple signaling pathways, including mitogen-activated protein kinase (MAPK), phosphoinositide kinase 3 (PI3K), and cyclooxygenase (COX) 2 pathways [25]. CB1R is mainly present in the brain; its particularly high levels are present in the hippocampus, cerebellum, and basal ganglia of the brain. In addition, it is worth mentioning that CB1R is also expressed in other sites such as peripheral sensory neurons, the immune system, the gastrointestinal tract, adipose tissue, the liver, the pancreas, reproductive organs, skeletal muscle, and cardiovascular tissues [26]. CB2R was initially found in immune cells and later also in the brain [27]. The interaction between the individual components of the ECS—endogenous ligands—N-arachidonoylethanolamine (anandamide, AEA) and 2-arachidonoylglycerol (2-AG), CB1, CB2 receptors, and metabolic enzymes (e.g., fatty acid amide hydrolase—FAAH and monoacylglycerol lipase—MAGL)—regulates the normal level of ECS activity, which is referred to as ECS tone and plays an important role in various pathophysiological mechanisms [28]. Numerous studies have shown that cannabinoids can also potentially interact with other non-CB1/non-CB2 receptors, including transient receptor potential cation channels subfamily V member 1—TRPV1, which is known as capsaicin or vanilloid receptor VR1, peroxisome proliferator-activated receptors (PPARs), and GPCRs such as GPR55 and GPR119 [29]. Due to these mechanisms, cannabinoid receptors and others are involved in many biological processes, such as the regulation of mood, cognitive function, nociception, appetite, lipid metabolism, cell growth, and proliferation [30], which is the focus of researchers’ studies.

The endocannabinoid system acts by increasing appetite and the motivation to seek food. This mechanism is regulated by food and anorexigenic transmitters, which are produced by the hypothalamus. These transmitters include, for example, corticotropin-releasing hormone (CRH), melanin-concentrating hormone (MCH), and hypocretin. Activation of the CB1 receptor signaling pathway can increase food absorption [31]. On the other hand, inhibition of ECS signaling weakens the mechanisms of food absorption, which results in a decrease in body weight [32]. Numerous studies have shown that the ECS regulates appetite centrally and peripherally, mainly by controlling leptin and ghrelin signaling [33,34]. Leptin negatively regulates CB1 receptor expression in the hypothalamus, and the common leptin resistance found in obese individuals impairs negative leptin control of ECS at the hypothalamic level, resulting in an increase in pro-nutritional behavior [35]. Administration of an inverse CB1 receptor agonist restores leptin sensitivity and as, shown in mouse studies, has an anti-obesity effect [36]. On the other hand, leptin reduces endocannabinoid synthesis by reducing intracellular calcium levels and glucocorticoid-mediated CB release [37]. It is worth mentioning that excessive activation of the central endocannabinoid system is associated with impaired leptin signaling, which contributes to the development of obesity [35]. In obesity, increased levels of leptin and decreased production of adiponectin, an anti-inflammatory hormone that protects against the development of insulin resistance and diabetes, have been observed [38]. Chronic CB1 activation may exacerbate processes associated with hyperlipidemia, diabetes, or cardiovascular events among hedonic obese patients [39], but impaired endocannabinoid signaling can result in eating disorders [40]. In patients with anorexia and bulimia nervosa, there is an increased level of CB1R mRNA in the blood [40].

**Figure 1:**
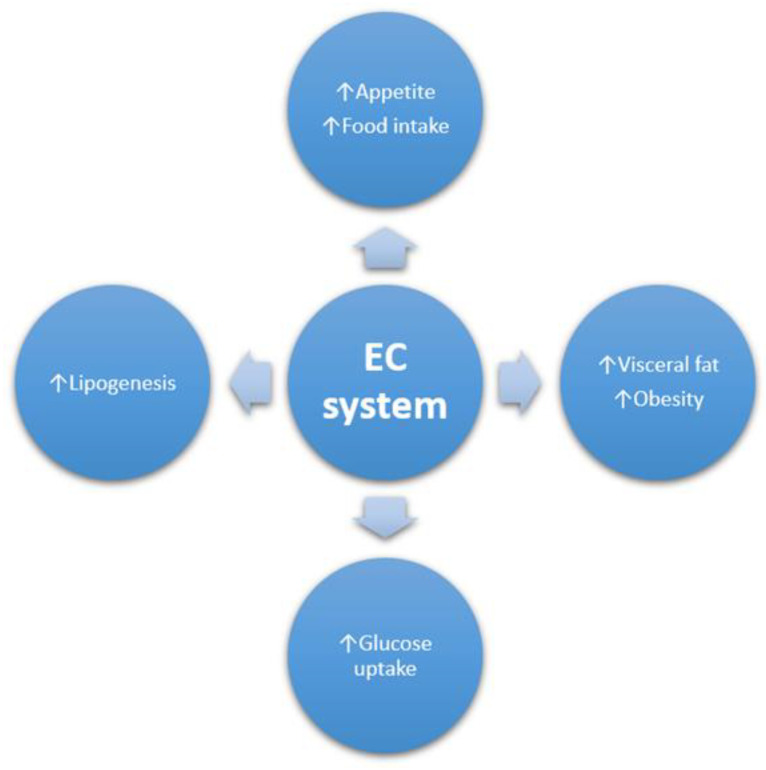
The role of the endocannabinoid system in the development of the metabolic syndrome.

There is a lot of scientific evidence showing a relationship between activity in the endocannabinoid system and the metabolic syndrome. Therefore, effective drugs that would affect this system are constantly sought. An example is rimonabant, a synthetic CB1R inverse agonist that has been shown to be an effective drug for reducing food intake and body weight. In addition, due to its mechanism of action, it alleviated insulin and leptin resistance caused by obesity. It was also effective in improving glucose hemostasis and dyslipidemia and contributing to the reduction of liver steatosis in obese and metabolic syndrome patients. Unfortunately, due to numerous side effects—anxiety, depression, suicidal thoughts—it was withdrawn from the market in 2009 [41]. This showed the need to search for drugs with a similar mechanism but that will not cause such severe side effects. As mentioned before, numerous studies over the past few years have proven that acute CB1 stimulation by cannabis extract initially increases food intake. In contrast, chronic consumption resulting in overstimulation of this receptor induces tolerance, which may be the result of CB1 internalisation [42]. Interestingly, studies have shown that human adipose tissue can respond to AEA and 2-AG due to the expression of two cannabinoid receptors [42]. The EC system also regulates adipose tissue function by promoting fat storage in adipocytes. This is due to the increase in adipogenesis and the increase in the production of triacylglycerols (TAGs) [43]. Why would inhibiting CB1 receptors be relevant in this case? Studies have shown that CB1R activation is associated with increased fat accumulation in adipose tissue. These receptors can increase the activity of lipoprotein lipase, the key enzyme that regulates the hydrolysis of triglycerides to free fatty acids, which are then deposited in the liver [44]. These processes, associated with the accumulation of lipids in the liver, can lead to the development of NAFLD, in which increased expression of cannabinoid receptors has been proven. It is worth mentioning that CB1R has been recognised as a key factor in the development of insulin resistance, increased lipogenesis, and liver steatosis, and CB2R is associated with the development of inflammatory processes in this last one [44]. Numerous studies have shown that CB1R antagonists reduce hyperglycemia and dyslipidemia. They also improve insulin resistance and glucose tolerance in obesity and type 2 diabetes [38]. The mechanism of action of cannabinoids, both exo- and endogenous, is closely linked to the EC system. Stimulation of the CB1 receptor activates the orexigenic pathway, resulting in increased appetite and stimulation of adipose tissue—this leads to lipogenesis and weight gain, resulting in obesity. In addition, activation of this receptor leads to an increase in plasma triglyceride and cholesterol levels, which is well known to be a factor in the development of liver steatosis and cardiovascular disease. CB1 receptors are also implicated in the development of insulin resistance and the risk of type 2 diabetes [38]. Figure 1 shows how important the endocannabinoid system is in terms of the risk of developing the metabolic syndrome.

Due to the different affinities of Δ9-THC and CBD for receptors in the endocannabinoid system, it can be assumed that they will exert different cellular effects. Δ9-THC has been shown to be a partial agonist of CB1 and CB2 receptors and binds to them with nanomolar affinity [45,46]. In contrast, CBD, with its micromolar affinity for cannabinoid receptors, is an inverse agonist of CB2R and a negative allosteric modulator of CB1R. Interestingly, CBD can antagonise the actions of CB1 and CB2 receptor agonists at concentrations lower—nanomolar—than those resulting from its affinity for these receptors [17]. Additionally, the presence of CBD in the endocannabinoid system can significantly attenuate the effects of Δ9-THC [47]. As mentioned before, cannabinoids can also interact with non-CB1/non-CB2 receptors. The multitude of these interactions with various receptors throughout the body proves the great potential of these substances in the diagnosis and treatment of many diseases and provides grounds for the search for optimal therapies. Examples of these receptors and the affinity of CBD and Δ9-THC for them are presented in Table 1.

**Figure 2:**
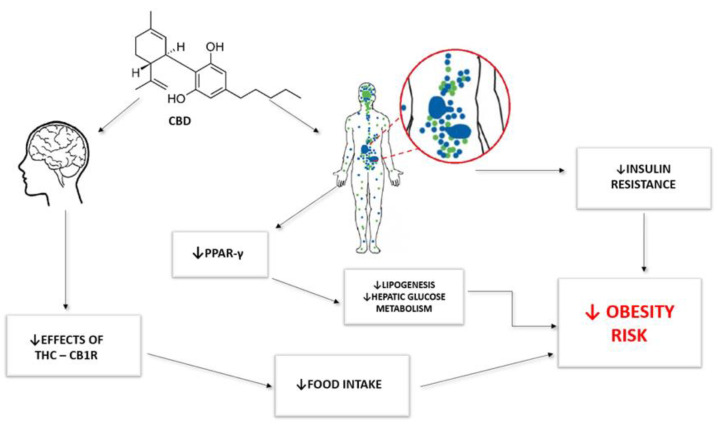
Mechanism of action of CBD in the potential treatment of obesity and related metabolic syndrome (Data from Fearby N., 2022 [47]).

Numerous studies confirm that Δ9-THC is a well-known appetite stimulant, and increased food intake leads to greater weight gain, which reduces cachexia. The results of clinical trials also support the efficacy of an oral cannabis extract containing THC/CBD for the prevention of refractory chemotherapy-induced nausea and vomiting [48]. Despite the long-known orexigenic properties of cannabinoids, in light of recent studies, CBD appears to have properties that reduce food intake [49,50,51]. Interestingly, studies on the endocannabinoid system and the control of food intake have shown that high doses of cannabinoids have orexigenic effects and low doses have anorexigenic effects [52]. This finding translates into the potential use of cannabinoids in the treatment of obesity-related conditions. Furthermore, there are scientific reports confirming the effect of CBD on vasodilation and its therapeutic effect on endothelial dysfunction, which may be due to diabetes or high glucose intake [53]. Thanks to its properties, CBD has an effect on the cardiovascular system, which is due to the activation of the TRPV channel, nuclear factor-kB (NF-κB), and map kinase (MAPK). By antagonising PPAR-γ, CDB increases available nitric oxide and lowers blood pressure [53]. Moreover, PPAR-γ plays a key role in the regulation of glucose homeostasis [54]. It is worth mentioning that CBD, as a functional antagonist of the GPR55 receptor, can control the release of pro-inflammatory cytokines such as IL-12 or TNF-α [53], which increase obesity and diabetes. Figure 2 shows how CBD may be related to various components of the metabolic syndrome. Δ9-THC, due to its psychotropic properties and stimulation of appetite (which promotes weight gain), resulting from the stimulation of CB1R in the CNS, seems to be a less important potential factor in the treatment of metabolic syndrome [55]. In addition, many researchers emphasise the need for further research on the impact of Δ9-THC on many diseases and rightly point out that there are many contraindications, mainly due to the psychogenic side effects of this substance, which may exclude Δ9-THC from potential use in some people.

**Table 1:** Examples of other receptors that cannabinoids can interact with (Data from Kicman A., 2020 [17] and Castillo-Arellano J., 2023 [56]).

Selected Phytocannabinoids	Role	Receptors
Cannabidiol (CBD)	Agonist or Positive allosteric modulator	TRPA, TRPV1/2, α1-GlyR, GABA_A_R, A_1A_R/A_2A_R, and 5HT_1A_R/5HT_2A_R.
	Antagonist or Inverse agonist or Negative allosteric modulator	5HT_3A_R, Nav_1_._1–1_._7_, GPR55, GPR18, GPR3, GPR6, GPR12, Kv2.1, TRPM8, Cav_3_._1–3_._3_, α7nACh, δ-OR, and µ-OR.
Δ^9^-Tetrahydrocannabinol (Δ^9^-THC)	Agonist or Positive allosteric modulator	GPR55, GPR18, PPAR-γ, TRPA1, TRPV2, 5-HT_2A_R, α1-GlyR, and α1β1-GlyR.
	Antagonist or Inverse agonist or Negative allosteric modulator	5HT_3A_R, δ-OR, and µ-OR TRPM8.

TRPA—transient receptor potential cation channel subfamily A, TRPV1/2—transient receptor potential cation channel subfamily V member 1/2, TRPM8—transient receptor potential cation channel subfamily M (melastatin) member 8, α1β1-GlyR—glycine receptor subunit alpha 1 and beta1, GABA_A_R—gamma-aminobutyric acid type A receptor, A_1A_R/A_2A_R—adenosine A1/A2 receptor, 5HT_1A_R/5HT_2A_R/5HT_3A_R—serotonin 1A/2A/3A receptor, Nav_1.1–1.7_—voltage-gated sodium channel, GPR55—G protein-coupled receptor 55, GPR18—G protein-coupled receptor 18, GPR3—G protein-coupled receptor 3, GPR6—G protein-coupled receptor 6, GPR12—G protein-coupled receptor 12, Kv2.1—potassium voltage-gated channel, Cav_3.1–3.3_—T-type calcium channel, α7nACh—alpha 7 nicotinic acetylcholine receptor, δ-OR—delta opioid receptor, µ-OR—mu opioid receptor, PPAR-γ—peroxisome proliferator-activated receptor gamma.

## 3. Methodology

The PubMed and Google Scholar databases were searched using combinations of the keywords: cannabis, cannabinoids, cannabidiol, metabolic syndrome, obesity, overweight, type 2 diabetes mellitus, glucose metabolism, dyslipidemia, low-density lipoprotein/LDL, triglycerides, hypertension, blood pressure, non-alcoholic fatty liver disease/NAFLD, liver steatosis. The search mainly included research published in the years 2007–2023.

## 4. Cannabinoids Relationship with Obesity

Obesity is an inseparable part of the metabolic syndrome. It is a basal condition from which other components of this disorder arise as a consequence of having too much body mass. Regardless of the definition used, it is associated with excessive fat accumulation and a chronic low-grade inflammatory state, which undoubtedly leads to further health implications [57]. As reported by the WHO, worldwide obesity has nearly tripled since 1975. The fast growth of obese people has made it a global problem, which is why new solutions to stop this trend are being sought all the time. The amount of food consumed is regulated both by central and peripheral mechanisms. Central mechanisms are related to eating behavior and feeling pleasure, while peripheral mechanisms are associated with adipogenesis, lipogenesis, glucose, and lipoprotein metabolism, in which ECS has a meaningful function [43].

A lot of studies provide evidence that cannabis use is related to increased calorie intake [58,59]. Surprisingly, some authors report that despite increased food consumption, cannabis users have a lower Body Mass Index [59,60]. As an explanation of this phenomenon, Clark et al. started from the fact that the American diet is proinflammatory, obesogenic, and increases the ratio of omega-6 (linoleic acid, LA) to omega-3 (α-linolenic acid, ALA) fatty acids. This increased ratio is the main cause of dysregulation of the endocannabinoid system because LA and ALA are precursors of substances that modulate CB1 and CB2 receptors. An increased level of these fatty acids leads to overstimulation of CBR1, which leads to excessive weight gain. According to its authors, the acute and long-lasting downregulation of CBR1, caused by reduced sensitivity for fatty acid metabolites following acute cannabis consumption, reduces energy storage and reverses the elevated fatty acid ratio’s impact on weight [59]. Interested in these data, we decided to look further for studies describing cannabidiol’s effect on the endocannabinoid system and how these mechanisms relate to obesity.

It is documented that CB1 and CB2 endocannabinoid receptors are located on subcutaneous and visceral adipose tissue, so they seem to be a promising target for cannabidiol. One of the studies was conducted on rats subjected to repeated intraperitoneal injections of CBD in doses of 2.5 and 5 mg/kg per day. Both doses caused a reduction in body weight in these rodents, which was more strongly expressed at the higher dose. A selective CB2 receptor antagonist was also used and found to have no effect on body weight on its own, but when administered with CBD, it reversed its effect on body weight. It can therefore be assumed that the effect of cannabidiol on body weight is due to its effect on the CB2 receptor [61]. However, the results of the other studies do not allow us to define an unambiguous position. Wierucka-Rybak et al. have provided research on rats divided into groups maintained on either a high-fat diet or a free-choice diet consisting of high-sucrose and normal rat food, which additionally received 3 mg/kg CBD. The results have shown that the rats on a high-fat diet and CBD injections consumed less food and presented an increased body weight; in turn, rats on a free-choice diet and CBD injections did not present a significant change in food intake or weight. In addition, there was another group of rats in which the effect of leptin on body weight gain in rats fed a high-fat diet was abolished by the simultaneous CBD injection [62,63].

We also found research where scientists investigated the attenuation of oxidative stress and the inflammatory response by chronic cannabidiol administration in rat skeletal muscles in a rat model of obesity. Neither rats on high-fat diets nor on standard food showed significant changes in body weight [64]. Therefore, it seems that we have another study where the effect of cannabidiol on body weight or food intake turns out to be different.

Intrigued by these results, we looked for other studies on the effects of cannabidiol on people’s body weight. Due to that, we analysed the crossover-designed clinical trial in which ten males were given CBD-rich Cannabis sativa (9% CBD, <1% THC). There was no notable difference in reported hunger, sweet food intake, or food preference between interventions, but compared to THC and placebo, after cannabidiol inhalation, participants presented a reduced desire to eat and a longer feeling of satiety [65].

In another trial with 62 participants with non-insulin-treated type 2 diabetes, orally administrated cannabidiol in doses of 100 mg twice daily for 13 weeks had no influence on appetite, body mass, or waist circumference [66]. Similarly, in the trial investigating the influence of CBD administration in COVID-19, the participants receiving cannabidiol in doses of 10 mg/kg per week or 20 mg/kg per week for two weeks did not present any changes in appetite or body weight compared to the placebo group [67].

Research and results on the effects of CBD on body weight are contradictory. Drawing conclusions from the above results, in our opinion, should be studied in order to establish the source of the conflict in the research studies provided above. Therefore, we refrain from forming any further conclusions pending further research on the effects of CBD on human obesity.

## 5. Cannabinoids Impact on Glucose Metabolism

Glucose metabolism disorders are closely related to the metabolic syndrome. This disturbs the homeostasis of the whole organism, which is why we are constantly looking for new solutions in the treatment of carbohydrate metabolism disorders and metabolic syndrome in general. The endocannabinoid receptors are located on adipose tissue cells and also on animal and human pancreatic islets [42]. Within the islets of Langerhans, there is an autonomous endocannabinoid system that modulates basal and glucose- and incretin-induced insulin secretion [68]. Many studies underline the role of the endocannabinoid system in the lipid signaling system, energy balance, and disorders within its modulation, which are considered to be related to the development of obesity and insulin resistance [69]. Cannabidiol, as a cannabinoid receptor modulator, impacts endocannabinoid system actions, regulates food intake and energy homeostasis, and results in body weight changes [43].

In one of the newest research projects from 2022, a group of scientists studied the effect of CBD on adipose tissue glucose uptake. They incubated isolated material with glucose and different concentrations of cannabidiol (30–240 µg/mL) in 37 °C for 2 h under 5% CO_2_ and 95% O_2_. The authors also formed two controlled groups incubated under the same conditions—the first of them was isolated tissue incubated with cannabidiol, and the second was isolated tissue without glucose and/or cannabidiol, but metformin was a standard drug in a dose of 240 µg/mL. In the results, there was a noticeable glucose uptake in adipose tissues incubated with cannabidiol and glucose in the study group. The results were dependent on dose, and when compared to metformin, they turned out approvingly. Analysing this and another piece of data that they received in the study, the authors suggest the potential of cannabidiol to maintain glucose-lipid homeostasis [70].

According to Cinar et al., the endocannabinoid system and the development of insulin resistance are related to excessive sphingolipid deposition. They conducted a study on obese mice and concluded that the cause of the above metabolic disorders must be found in the activation of the CB1 receptor in hepatic cells, which increased ceramic de novo synthesis and insulin resistance in hepatic cells [71]. What is interesting is that in one of the newest studies from 2022, researchers have a similar opinion. They explored how intraperitoneal cannabidiol injections in an animal insulin resistance model affect sphingolipid metabolism and result in insulin resistance in subcutaneous and visceral adipose tissue. The results proved that in conditions of increased free fatty acid intake in high-fat diet-fed male Wistar rats, CBD sensitises adipose tissue to insulin due to its influence on sphingolipid metabolism. The authors drew the conclusion that cannabidiol may be a potential target for reducing insulin resistance, type 2 diabetes mellitus, and the metabolic syndrome [72].

Another study describes how chronic CBD administration in obese rats influences bioactive lipids and glucose metabolism in red skeletal muscles. Using immunoenzymatic and colorimetric methods, they marked glucose and insulin concentrations after a 10 mg/kg CBD injection for two weeks. They concluded that CBD administration decreased the intramuscular de novo ceramic synthesis pathway in the obese animal model and increased other sphingolipids like sphingosine-1-phosphate, which improved insulin transduction and glycogen recovery in the skeletal muscle of the obese animal model [73].

As for the works in which the authors strictly referred to the metabolic syndrome, we found a study about how THC and CBD affect metabolic syndrome parameters, including the microbiome, in mice fed a high-fat, high-cholesterol diet. It turned out that rats on this diet that additionally received CBD had reduced fasting glucose levels and lower glucose levels at the 120 min oral glucose tolerance test compared to rats on the same diet that did not receive cannabidiol [74]. Due to the above, it seems that CBD, presenting an influence on glucose, could be a potential target in glucose homeostasis disorders, even drawing bolder conclusions in metabolic syndrome, because the authors came to the conclusion that CBD also improved lipid parameters [74].

However, data on cannabidiol and its impact on glucose metabolic processes are ambiguous. Khalid et al. conducted double-blind, placebo-controlled research on tetrahydrocannabivarin and cannabidiol’s influence on 62 subjects aged ≥18 with non-insulin treated type 2 diabetes. Three months before research, subjects needed either to receive no oral hypoglycemic drugs or take stable doses of metformin, sulfonylurea, dipeptidyl peptidase-4 inhibitor, or glucagon-like peptide one. Researchers have based their data on data obtained from standard 75 g OGTT, plasma glucose, serum insulin levels, insulin sensitivity, and B-cell function calculated on the HOMA2 calculator. They concluded that there was no significant difference in glucose response to OGTT at 2 h. in patients with only CBD substitution and that CBD did not produce any improvement in glycemic control compared with placebo. What is interesting are the much more promising results given by THVC substitution, but that is not the subject of this review [66].

Other scientists conducted a double-blind, placebo-controlled study based on applying two oral CBD formulations. One of them, except for CBD (45 mg), contained American ginseng, ginkgo biloba, and organic hemp oil; the second preparation was CBD encapsulated in 150 mg hemp oil. Neither of these two formulations has been shown to affect glucose or insulin levels [75]. These should be compared to findings from one of the newest research projects conducted on a group of males with ≥25 kg/m^2^ body mass index. Consumption of a mixed macronutrient meal and administration of CBD did not influence the glucose response to food in comparison to placebo; however, CBD evoked lower insulin concentrations in males with overweight and obesity compared to placebo [76]. If the above results are considered in the context of metabolic syndrome, they seem interesting.

To have an even more ambiguous opinion, we found another piece of research whose findings suggest that fetal exposure to cannabis is associated with increased adiposity and fasting glucose levels in the first years of life [77]. For this purpose, the subsamples of 103 mother-child pairs were examined. Not all of them were exposed to cannabinoids during pregnancy. Compared to nonexposed offspring, exposed offspring had about 8 mg/dl higher fasting glucose levels at age 4.7 years. It has not been proven that it had any influence on HOMA-IR, which reflects insulin resistance in children [78]. Twelve cannabinoids or their metabolites were detected in the urine samples of 27 week old pregnant women. THC and its derivatives were the most often detectable substances, and others, which were much less detectable, were CBD, CBC, CBN, CBG, and CBDV. In our opinion, this would mean that studies should be carried out on how individual compounds affect fetal metabolism in order to have a real result [77].

Therefore, the results of these and the previously described study seem to be consistent, but their conclusions are in stark contrast to the research described at the beginning of this subsection. It means that there could be another factor that changes how cannabidiol acts on carbohydrate metabolism, and more research is needed to know exactly what the effect of CBD on glucose metabolism and other processes related to diabetes is.

## 6. Plasma Lipid Level and Cannabis

One of the components of the metabolic syndrome is atherogenic dyslipidemia. It is defined as the coexistence of elevated TG concentrations, decreased HDL-C concentrations, and the presence of abnormal (small, dense) LDL particles. Statins are currently the mainstay and first-line treatment for most lipid disorders, as this group of drugs has a number of pleiotropic effects and has been shown to reduce mortality.

In recent years, there have been an increasing number of studies investigating the effects of cannabis-derived compounds on lipid metabolism (Table 2). Some of these reports suggest that this class of preparations may have a favorable effect on the lipid profile, thereby improving the overall metabolic condition of the patient. Given the increasing availability of such products on the market, the evaluation of the effects of hemp derivatives on lipid metabolism appears to be important from a public health perspective.

Some in vitro studies have shown promising results with cannabis oil derivatives in the treatment of hypercholesterolemia. The hypocholesterolemic effect of hempseed protein hydrolysate was first suggested in 2017 [79]. The study conducted by Zanoni et al. showed that hempseed peptic hydrolysate was able to inhibit HMG-CoA reductase both directly and by increasing its phosphorylation. In addition, the authors indicated that certain hempseed peptides have the potential to upregulate LDL receptors via elevating SREBP2 protein, leading to enhanced LDL uptake by hepatocytes. Interestingly, this mechanism is similar in some respects to the mechanism of action of statins. In addition, this study also showed an increase in PCSK9.

A promising starting point for the synthesis of new lipid-lowering compounds may be hempseed peptide H3 (IGFLIIWV), a product of C. sativa hemp oil hydrolysis [80]. It has been shown in in vitro hepatocyte models to enhance cholesterol-lowering effects, particularly by modulating PCSK9 activity. Some reports suggest that the effects of hemp derivatives may be diminished by concomitant statin therapy [81], so possible adjunctive therapy does not seem warranted. Another study investigated how the administration of CBD preparations to young rats can affect the activity of various metabolic parameters and antioxidant activity later in life [82]. The study showed that CBD administration during adolescence can significantly modulate the body’s metabolic status later in life. Significant reductions in plasma, white adipose tissue, brown adipose tissue, and liver TG levels were observed in adult rats that received CBD injections during adolescence.

Animal model studies support the concept that a cannabis-containing diet may ameliorate manifestations of hypercholesterolemia [83]. Hempseed-based diets significantly improved lipid profiles in Wistar rat models of hypercholesterolemia. A significant reduction in TC, LDL, and TG and a concomitant increase in HDL were observed compared to the control group.

There are also reports that hemp oil-based formulations markedly reduced the severity of atherosclerotic lesions in mouse models [84]. Probably it is due to CBD and THC’s ability to attenuate TNF-α elevation observed in patients with metabolic disorders, and as we know, TNF-α seems to be a prominent pro-inflammatory cytokine and factor promoting insulin resistance [85]. Among CBD derivatives, cannabidiolic acid (CBDA) seems to be an interesting compound for the treatment of metabolic disorders. CBDA itself is a rather unstable particle, so in order to stabilise CBDA, a new derivative, CBDA-O-methyl ester (HU-580, EPM301), was synthesised [86]. EPM301 improved the serum lipid profile in high-fat diet-induced obese mice, as manifested by reduced serum TG and total cholesterol. EPM301 also increased the HDL/LDL ratio without altering HDL levels, but reduced LDL levels. In addition, amelioration of HFD-induced liver injury and steatosis was observed.

Can smoking marijuana have a beneficial effect on lipid parameters? A study conducted in Peru evaluated TC, HDL, and LDL levels in a group of young people who declared to smoke marijuana frequently [87]. Measurements were taken 30 and 120 min after smoking. A statistically significant increase in HDL was observed at 120 min. A review by Lazarte et al. also suggests a weak association between cannabis smoking and beneficial effects on the lipid profile. However, this relationship needs further verification [88].

Alonso et al. analysed risk factors for metabolic syndrome in patients with newly diagnosed psychotic symptoms during a one year follow-up period [89]. Among patients who reported regular cannabis use, a negative correlation between cannabis use and fasting TG levels was observed, but it was not statistically significant. After a period of 1 year, a significant positive correlation was found between HDL levels and declared cannabis use. On the other hand, a similar study assessing patients with schizophrenia who declared cannabis use in a one year follow-up period showed some favourable changes in metabolic syndrome parameters but without statistical significance except for the change in waist circumference [90]. Another study showed an inverse relationship between cannabis use and the prevalence of metabolic syndrome in young patients with a first episode of psychosis [91]. Furthermore, cannabis use was associated with considerably lower triglycerides.

Afshar et al. evaluated the effect of administration of a sublingual formulation containing CBD and THC at a ratio of 10:1 in a group of 50 patients with type 2 diabetes [92]. After 8 weeks of treatment, reductions in TC, TG, LDL, and HDL were observed compared to the placebo group, and the differences were statistically significant for the first three parameters. Interestingly, the researchers also suggest that the sublingual route of administration may significantly improve the bioavailability of cannabinoids compared to per os administration because of the diminished first-pass effect.

The other study looked for correlations between cannabis use and components of the endocannabinoid system in people over the age of 60 [93]. Researchers investigated how short-term administration of exogenous cannabinoids can modulate the balance of the body’s endogenous cannabinoid system. Although no statistically significant effect of cannabis use on the evaluated lipid parameters was observed, the study confirmed a significant positive correlation between 2-AG (2-arachidonyloglicerol) and TG levels, which has been reported in other papers before [94].

Some data implies CBD preparations may lower postprandial triglycerides. One article showed a statistically significant reduction in TG levels between the CBD group and the placebo group in measurements performed 30 min after ingestion of CBD [76]. The mechanism of this phenomenon remains unclear, as the measured concentrations of CBD and its metabolites remained relatively low in the early postprandial period.

It is important to emphasise that many of the studies to date have been conducted in animal models, and there is certainly a lack of large studies evaluating the clinical efficacy of hemp-derived compounds. Furthermore, the aforementioned clinical trials have several limitations, such as the small number of patients enrolled, the usually non-standardised dosing pattern, and the presence of many confounding factors. Taking this into account, conclusions about the effect of cannabis compounds on lipid parameters should be drawn with caution. Further research is desirable.

**Table 2:** Comparison of selected studies (DM2—diabetes mellitus type 2, HMG-CoAR—3-hydroxy-3-methyl-glutaryl-coenzyme-A reductase, PCSK9—proprotein convertase subtilisin/kexin 9, HT—hypertension, PD—Parkinson’s disease, OEA—oleoylethanolamide, PEA—palmitoylethanolamide).

Authors	Characteristics of the Study Group	Cannabis Formulation Characteristics	Statistically Significant Changes in Lipid Metabolism Parameters	Duration of the Observation	Reference
Zanoni et. al	Human hepatic HepG2 cells, in vitro study.	Ninety different peptides are derived from Cannabis sativa hydrolysis.	Inhibition of HMG-CoAR activity ↑LDLR expression ↑PCSK9.	-	[79]
Afshar et al.	DM2, mean age 55.7 years old.	Sublingual spray CBDEX10 twice daily (200 µg/20 µg CBD/Δ9-THC).	↓TC, ↓TG, and ↓LDL.	8 weeks.	[92]
Abuhasira et al.	HT, PD, and mean age 69 years old.	Herbal cannabis supplements with different CBD:THC ratios and different dosing patterns.	Positive correlation of 2-AG and TG change and negative correlation of HDL/LDL ratio and OEA and PEA.	3 months.	[93]
Cusihuaman et al.	Mean age 31 years old, otherwise healthy, declared daily-smokers of marijuana for at least 12 months	Approximately 0.2 g of *Cannabis spp.* administration by the pyrolytic route.	↑HDL at 120 min.	Measurements at 30 and 120 min after smoking.	[87]
Li et al.	human hepatic HepG2 cells: an in vitro study.	*Cannabis sativa* Peptide H3 (IGFLIIWV)	Inhibition of HMGCoAR activity, ↑LDLR expression, and ↓PCSK9.	-	[80]
Huang et al.	Animal model of atherosclerosis: apoE-deficient mice fed with a high-cholesterol diet.	cannabis seed oil; 30 μL by gavage using a micropipettor per day. (7.4% of palmitic acid, 3.0% of stearic acid, 10.8% of oleic acid, 55.8% of linoleic acid, 14.0% of α-linolenic acid, and 2.5% of γ-linolenic acid).	↓TG ↓LDL at week 6.	Measurements at weeks 4, 6, and 8.	[84]
Abbotts et al.	Mean age 26 years old, mean BMI 29.7.	Five different formulations, each containing 30 mg of CBD; seven doses (2 followed by a meal, five unrelated to the meal).	↓postprandial TG at 30 min.	Series of measures up to 240 min after CBD ingestion.	[76]
Alonso et al.	Schizophrenia.	Declared daily cannabis use.	Positive correlation between cannabis use and HDL.	One year follow-up.	[89]
Reyes-Cuapio et al.	Juvenile Wistar rats at 30 postanal day	CBD intraperitoneal injections (5, 10, or 30 mg/kg).	↓TG	14 days.	[82]
Kaushal et al.	animal model of induced hypercholesterolemia. Wistar rats fed with high-fat diet.	Special composition of hempseed diet for 1 or 2 months.	↓TG, ↑HDL, ↓LDL, and ↓TC.	1 or 2 months.	[83]
Ben-Cnaan et al.	Mice models of diet and genetic induced obesity.	CBDA-O-methyl ester (HU-580, EPM301) 40 mg/kg/day, intraperitoneal administration.	↓TG, ↓TC, and ↑HDL/LDL.	28 days.	[86]
Stiles et al.	Mean age: 23.4 years old, first episode of psychosis.	Analysis of data obtained from RAISE-ETP study.	↓TG.	24 months.	[91]

## 7. Cannabinoids Effects on Blood Pressure

Hypertension is another component of the metabolic syndrome of great importance, not only in the syndrome itself but also in other cardiovascular diseases like myocardial infarction, stroke, or heart failure [95]. Due to the wide dissemination of this disease entity, researchers have focused on the influence of cannabinoids, particularly CBD and Δ9- THC, on blood pressure and blood vessels. Data on the effect of cannabidiol on blood pressure are limited. We summarised the studies that were conducted on both animal models and human volunteers and obtained a wide spectrum of results.

Baranowska-Kuczko et al. constructed a study in which they evaluated the effect of CBD on the vascular endothelium (aorta and small mesenteric vessels) in rats. Some of the tested subjects had spontaneous hypertension (the SHR model of primary hypertension), and some had deoxycorticosterone-induced hypertension (the DOCA-salt model of secondary hypertension). A total of 10 mg/kg of cannabidiol (CBD) was administered daily for two weeks to the vessels of the rats mentioned above. Vasodilation enhancement was prevented throughout the study with NG-Nitro-L-arginine methyl ester (L-NAME) and/or the COX-1 and COX-2 inhibitor indomethacin. The results of the study indicate that a constant dose of CBD had a protective effect on vessels, reduced vascular hypertrophy, and stimulated nitric oxide (NO)-dependent arterial vasodilation. This may lead to the conclusion that CBD, by increasing the secretion of nitric oxide (NO) and thus leading to vasodilation, will lower blood pressure. In addition, the study did not show any harmful effects of daily use of a constant dose of cannabidiol on blood vessels, and its positive effects may in the future be considered for the treatment/prophylaxis of people with hypertension [96].

Sultan S et al. randomised, double-blind, placebo study conducted in 26 healthy men. Volunteers were exposed to 600 mg of CBD or a placebo. During the course, the effect of acute and repeated administration of cannabidiol on blood pressure was also studied. BP and other parameters, including HR, stroke volume (SV), cardiac output (CO), stroke time (ET), and total peripheral resistance (TPR), were measured at rest and during isometric exercises. Both acute and repeated CBD dosing showed no significant reduction in either systolic or diastolic blood pressure, but an immediate and significant decrease in mean arterial pressure (MAP) was demonstrated. The best effect of CBD on systolic blood pressure was revealed during isometric exercises that simulated a stressful situation. The results showed that in subjects exposed to CBD after 7 days of testing, SBP was on average −8 mmHg lower than placebo [97].

Khalid a Jadoon et al., with their randomised study, were aiming to investigate how CBD influences the cardiovascular response to stress. The response was tested after exposure to the stress factor by measuring blood pressure. On the day of the visit, participants were given a 600 mg dose of cannabidiol, and two hours later they performed cardiovascular stress tests (mental, isometric, and cold pressor stress tests), which showed that one dose of CBD reduces the cardiovascular response to stress by lowering blood pressure. The effect of CBD on resting parameters showed that CBD reduced systolic blood pressure by −6 mmHg but did not affect diastolic blood pressure or MAP. In the mental stress test and isometric exercises, the CBD-treated group showed lower blood pressure results compared to the placebo. The obtained results showed lower cardiovascular parameters, including blood pressure, not only during the test but also during the recoalescence period. During exercise stress, SBP or MAP were −5 mmHg lower than placebo. The cold pressor test has also been studied. During the course, an even increase in blood pressure parameters was observed. An interesting fact turned out to be the point at which the blood pressure parameters continued to increase in the placebo group and plateaued in the CBD study group. In addition, SBP and MAP values were on average −8 mmHg lower than in the placebo group. As in previous tests, blood pressure was also lower during rest after the stressor [98]. The study was not constructed to explore the effect of CBD on patients with hypertension because the subjects were healthy, but it shows that cannabidiol has a potential influence on blood pressure. Although the results of this study might be considered in later studies for patients with hypertension or metabolic syndrome.

Batinic A. et al. published in 2023 one of the latest studies showing the effect of cannabidiol on blood pressure. A randomised, double-blind, placebo-controlled study on a new oral form of CBD—DehydraTECH™ 2.0 CBD (Lexaria Bioscience Corp., Kelowna, BC, Canada). The study group consisted of volunteers with grade 1 or 2 hypertension. After taking a dose of DehydraTECH™2.0 CBD every 10 min for 180 min, blood pressure was measured and compared to a generic CBD control. The results of the study show that DehydraTECH™2.0 CBD reduces both diastolic and mean arterial pressure (MAP) and reduces heart rate (HR). The study proves that cannabidiol not only may have a reasonable effect in patients with hypertension but also that the search for new, better assimilable and bioavailable forms, such as DehydraTECH™2.0 CBD will expand the possibilities of using cannabidiol and/or increase its therapeutic effectiveness [99].

The above studies prove that the use of CBD in hypertensive patients can have positive effects. They will not be directly related to lowering blood pressure because the hypotensive effect of cannabidiol is small. However, CBD can be used to prevent pressure surges, stabilise them, and have a protective effect on blood vessels. Studies also show that CBD has a hypotensive effect, but it is visible under certain conditions (e.g., stress). This fact opens further possibilities for studying the effect of CBD on the cardiovascular system, including blood pressure and the metabolic syndrome itself. The effects achieved may also suggest the use of CBD as a treatment for hypertension. More research needs to be performed to develop the topic.

## 8. Non-Alcoholic Fatty Liver Disease and Its Correlation with Cannabis

Non-alcoholic fatty liver disease (NAFLD) is a chronic inflammatory disease of the liver closely related to the metabolic syndrome. In recent years, the influence of NAFLD has been proven not only on the liver but also on the systemic one. By acting on regulatory pathways, it increases the risk of type 2 diabetes, cardiovascular disease, and chronic kidney disease (CKD) [100].

Studies conducted on animal models allow us to assess the impact of cannabinoids on the metabolism of the liver affected by NAFLD. The high-fat cholesterol diet (HFCD) was used as a model for NAFLD because it induced inflammation, fatty liver, and increased liver enzymes (alanine aminotransferase (ALT), triglycerides, and total cholesterol) over a 6 week period. During the study, it turned out that under the influence of CBD, HFCD mice consumed larger amounts of food than other models. However, this was not followed by changes in body weight, body fat, or liver, as seen in the effect of CBD on metabolism. The main point of the study remained unaffected by administering CBD. Both cannabidiol and THC did not reduce fatty liver disease or improve liver function. After expanding the research, the supply of CBD may be beneficial, as it has been shown to reduce inflammatory parameters such as TNF-α and iNOS [74].

Administration of CBD in NAFLD is based primarily on the anti-inflammatory effect that cannabidiol presents. In mouse models, a reduction in the risk of developing NAFLD was observed with the use of CBD. This positive effect may be seen in the regulation of inflammasome pathways (NF-κB and NLRP3) [101].

Silvestri C. et al. studied the effects of cannabidiol (CBD) and Δ9-tetrahydrocannabivarin (THCV) on animal models and human hepatocyte line 5 (HHL-5) cell lines. The results of the study show that under the influence of CBD, there is a decrease in intracellular lipid levels in the tissue. The above-mentioned results show that the effect of cannabidiol on NAFLD may be potentially beneficial in the treatment of metabolic syndrome and its components. However, more research needs to be performed [102].

Due to the increasing incidence of NAFLD, the increased use of cannabinoids in society, and the constant search for therapeutic possibilities seen in Cannabis sativa derivatives, there will be further observations about the impact of CBD and other derivatives on the body and diseases. More human studies are needed to explore new therapeutic options and the effects of CBD on NAFLD. What seems to be beneficial is the fact that no significant side effects of cannabidiol have been observed. Because of the limited number of human studies, more research needs to be performed.

## 9. Conclusions

Despite the rapid growth of the cannabis derivatives business, this area is still understudied. Its application to the metabolic syndrome requires further study. The majority of the referenced research had a number of limitations, making it difficult to draw clear conclusions that can be transferred into clinical practice.

A substantial percentage of studies rely on animal models, making it unclear whether the findings can be applied in a clinical setting. Furthermore, most patient studies conducted to date have analysed relatively small groups of individuals. Additionally, the method of administration and dosage of CBD products vary greatly between studies. The lack of consistency in preparation makes comparing results difficult. Despite the aforementioned concerns, CBD preparations appear to have considerable future promise as an adjuvant treatment, considering all the different elements of the metabolic syndrome.

## Data Availability

Not applicable.
